# Kinetics of neutralizing antibodies against SARS-CoV-2 infection according to sex, age, and disease severity

**DOI:** 10.1038/s41598-022-17605-1

**Published:** 2022-08-05

**Authors:** Yoonjung Kim, Joon-Yong Bae, Kitae Kwon, Hyun-Ha Chang, Won Kee Lee, Heedo Park, Jeonghun Kim, Isaac Choi, Man-Seong Park, Shin-Woo Kim

**Affiliations:** 1 Department of Internal Medicine, Kyungpook National University Hospital, School of Medicine, Kyungpook National University, 130, Dongdeok-ro, Jung-gu, Daegu, 41944 Republic of Korea; 2 Department of Microbiology, Institute for Viral Diseases, Biosafety center, College of Medicine, Korea University, Seoul, Republic of Korea; 3 Department of Medical Informatics, School of Medicine, Kyungpook National University, Daegu, Republic of Korea

**Keywords:** Microbiology, Diseases, Medical research

## Abstract

Knowledge of the factors affecting the difference in kinetics and longevity of the neutralizing antibody (nAb) response to SARS-CoV-2 is necessary to properly prioritize vaccination. In the present study, from March to December 2020, of the 143 patients who recovered from COVID-19, 87 underwent study visits scheduled every 3 months. Patient demographics and blood samples were collected followed by a plaque reduction neutralization test to analyze nAb titers. A linear mixed model was used to compare the effects of sex, age, and disease severity over time. Results demonstrated a gradual reduction in nAb titers over time with a significant decrease from 6 to 9 months post-COVID-19 infection (*p* < 0.001). In time-to-sex, age, and disease severity comparisons, reduction in nAb titers over time was unaffected by sex (*p* = 0.167), age (*p* = 0.188), or disease severity (*p* = 0.081). Additionally, the nAb titer was 1.46 times significantly higher in those aged ≥ 50 years than in those aged < 50 years (*p* = 0.036) irrespective of time Moreover, the nAb titer was 2.41 times higher in the moderate or above than that in the below moderate disease severity group (*p* < 0.001). However, no significant differences were observed in terms of sex (*p* = 0.300). Given the reduction in nAbs over time, maintaining protective neutralizing antibodies regardless of sex, age, or disease severity is needed.

## Introduction

Severe acute respiratory syndrome coronavirus 2 (SARS-CoV-2) neutralizing antibodies (nAbs) are key to protection against COVID-19. Along with research on the rapid detection of COVID-19^1,2^, the population immunity developed through natural infection, or preferably through vaccination, is essential for combating the COVID-19 pandemic.

Currently, vaccination is administered worldwide. Although their global supply is limited, it is important to properly select and inject vaccines for the prevention of COVID-19. Vaccines are reported to have an effective protection rate of 50–95%^3^. In a recent study, nAbs strongly correlated with vaccine-induced immunity in humans^4^. Previous studies have confirmed the production of protective nAbs, which may prevent reinfection, in recovered patients. According to a retrospective study, the protection acquired from prior and symptomatic infections were 81.8%, and 84.5%, respectively^5^. Another study reported that a previous SARS-CoV-2 infection reduced the reinfection and symptomatic infection risk by 84% and 93%, respectively^6^. Additionally, a ferret animal study showed that nAb titer less than 20 resulted in reinfection^7^. Moreover, recovered patients showed diverse nAb titer ranges^8^. However, the duration and range of nAb titers required to prevent reinfection through natural and vaccine-acquired immunity in humans remain unknown. Therefore, further research on vaccine kinetics and duration of nAbs required in recovered patients is desirable.

Reportedly, a peak in nAb levels at around 4–5 weeks after the onset of symptoms is observed^9^, which gradually decreases over the next 3 months^10^. In addition, sex, age, and disease severity are known to affect nAb responses^11^. However, to date, few studies have investigated the differences in nAb titers over time based on sex, age, and disease severity.

Therefore, in the present study, we longitudinally evaluated and compared the nAb response according to sex, age, and disease severity in asymptomatic and critically hospitalized individuals infected with SARS-CoV-2.

## Results

**Figure 1:**
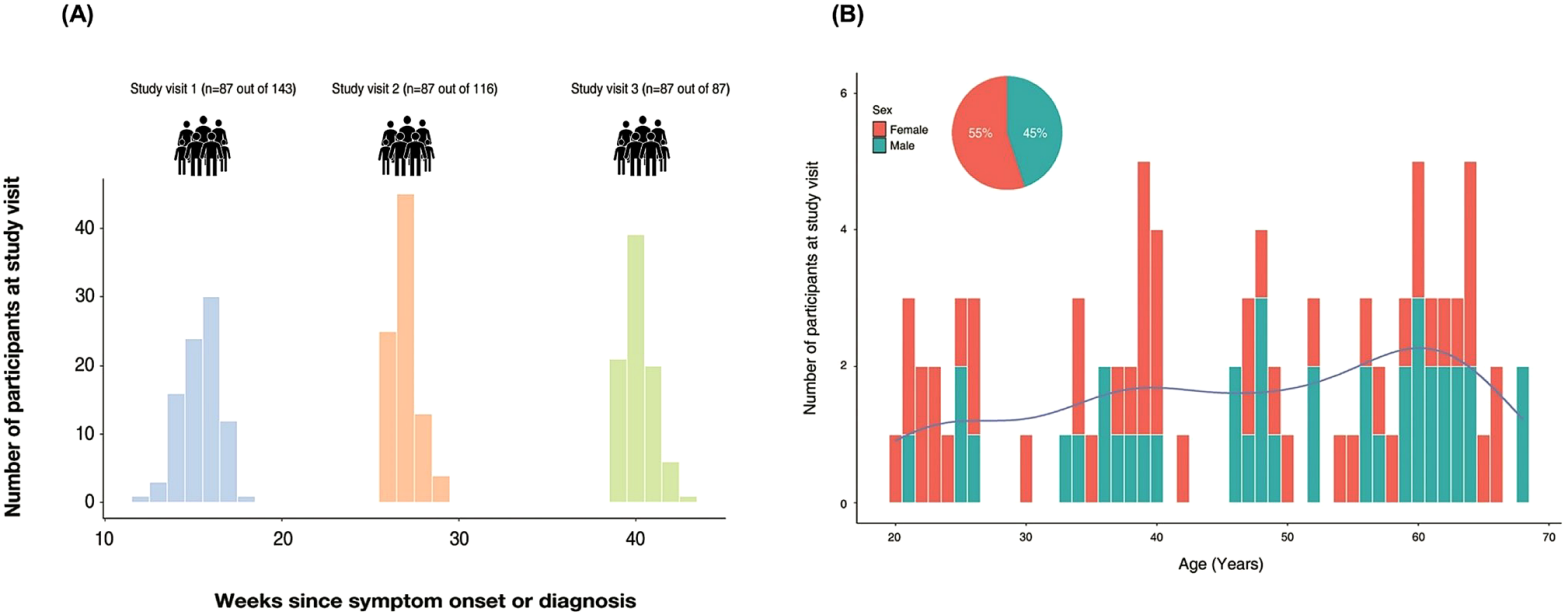
Study design and characteristics of study participants. (**a**) Study timeline and number of participants at each study visit. The graph represents blood sample collection time for participants from symptom onset or COVID-19 diagnosis (**b**) Participants’ age and sex distribution.

Of the 143 patients, 56 were excluded due to non-completion of three visits, and 87 were found eligible for the analysis (Fig. 1a). The median age was determined to be 48.0 years (IQR, 36–60 years), and 48 (55.2%) of the total patients were females (Fig. 1b). Regarding the disease severity, 51 (58.6%) patients were classified as mild, 20 (23.0%) as moderate-severe, 7 (8.0%) as severe, and 3 (3.4%) as critical patients. The mean interval time between the first and second visit was 111 days (range 105.5–116 days). The second and third time points showed a mean of 81 (range 76–85 days) and 92 days (range 91.5–93.5 days) after symptom onset, respectively.

### Persistence of natural immunity

**Figure 2:**
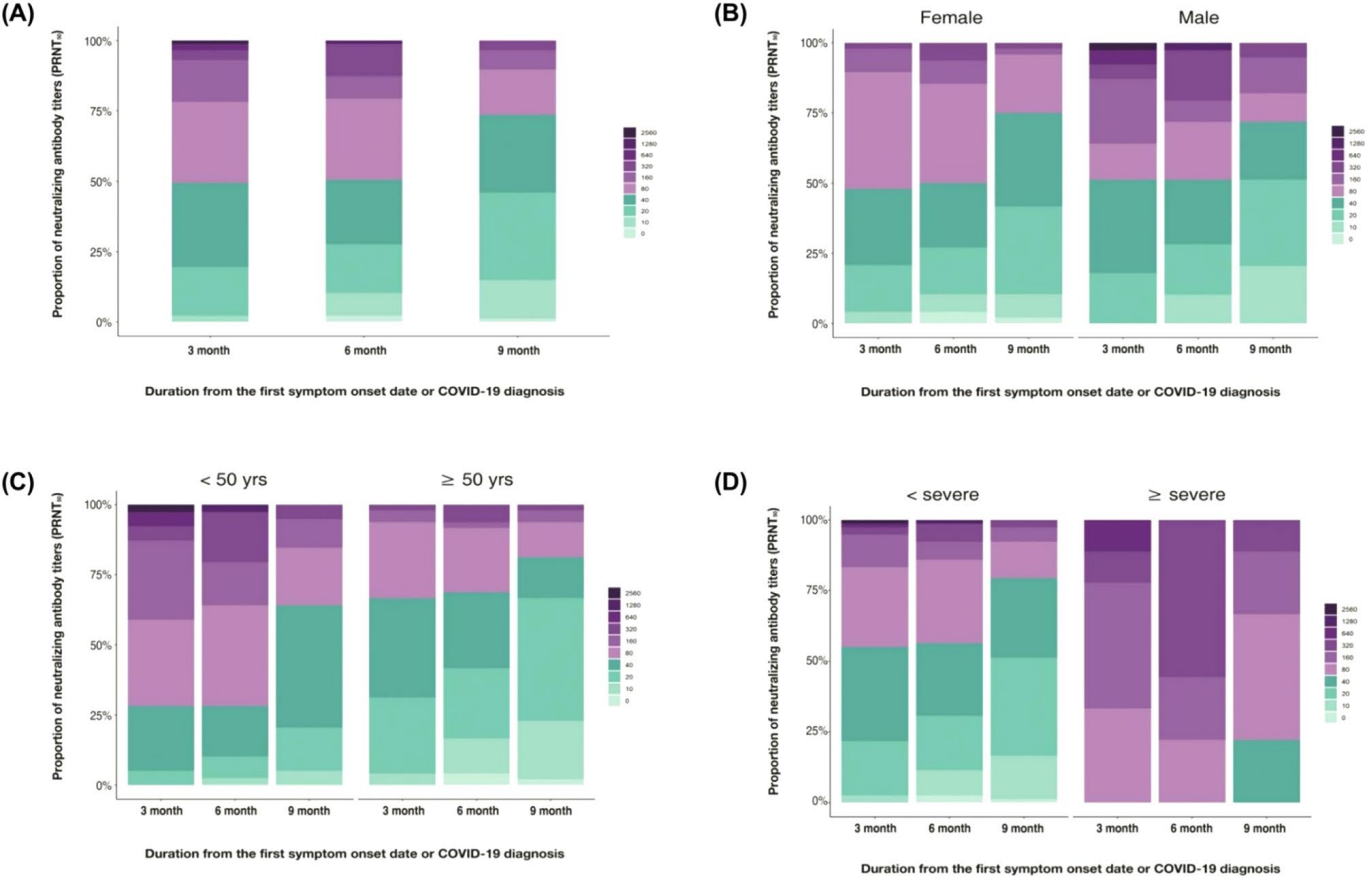
Distribution of SARS-CoV-2 neutralizing antibody (nAb) titers based on sex, age, and disease severity at intervals of 3 months. (**a**) The proportion of individuals with antibody titers ranging from − 2560 over the time points of 3, 6, and 9 months from the first COVID-19 symptom onset or diagnosis. (**b**) The proportion of individuals with antibody titers according to sex at the time points of 3, 6, and 9 months from the first COVID-19 symptom onset or diagnosis. (**c**) The proportion of individuals with antibody titers according to age < 50 or ≥ 50 years at the time points of 3, 6, and 9 months from the first COVID-19 symptom onset or diagnosis. (**d**) The proportion of individuals with antibody titers according to disease severity of < severe or ≥ severe at the time points of 3, 6, and 9 months from the first COVID-19 symptom onset or diagnosis.

**Figure 3:**
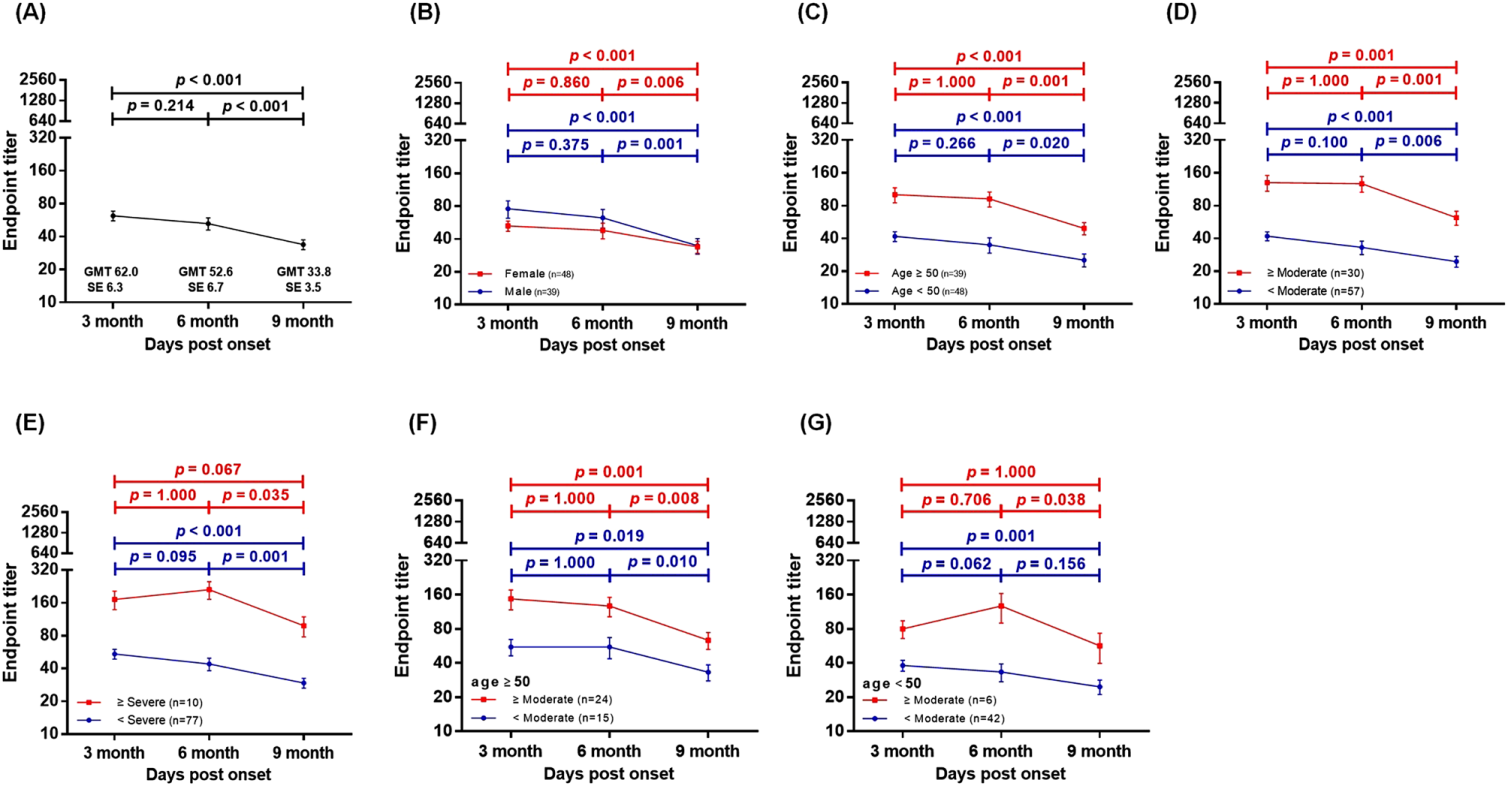
Variations of the neutralizing antibody (nAb) titer based on sex, age, and disease severity at intervals of 3 months. Titers are graphed as geometric mean titers (GMT) with geometric standard error. The *p*-values were obtained from repeated analysis of variance (ANOVA) measurements (**a**) Titers of 87 individuals who underwent blood sample collection after 3 months of COVID-19 symptom onset or diagnosis with subsequent sampling at 6 and 9 months (**b**) nAb titer variation between females and males. (**c**) nAb titer variation according to age between < 50 and ≥ 50 years. (**d**) nAb titer variation according to disease severity between < severe and ≥ severe disease severity. (**e**) nAb titer variation according to disease severity between < moderate and ≥ moderate disease severity. (**f**) nAb titers in ≥ 50 years age group according to moderate disease severity. (**g**) nAb titers in < 50 years age group according to moderate disease severity.

The nAb titers varied at each of the three different time points, and the kinetics of the nAb titers showed a decreasing tendency over time by the linear regression model (Supplementary Fig. 2). Patients with high nAbs titers showed a substantial robust persistence after 3–6 months of symptom onset or diagnosis; however, it decreased after 9 months (Fig. 2a). The comparison of all nAb titers revealed that GMT values at the first (3 months), second (6 months), and third (9 months) time points after COVID-19-related symptom onset or diagnosis were 62, 52.6, and 33.8, respectively. The GMT between the first and second time points showed a decreasing trend; however, the difference was not statistically significant (*p* = 0.214). In contrast, GMT at the third time point showed a significant reduction (*p* < 0.001) as compared to that at the second time point (Fig. 3a).

### Impact of sex difference on nAb kinetics

The proportion of female and male patients with high nAb titers was reduced over time (Fig. 2b). The GMT values for female patients at three time points were 52.6, 45.8, and 33, respectively, whereas those for male patients were 75.8, 62.4, and 34.7, respectively. Additionally, the kinetics of nAb responses in both female and male patients showed a non-significant decreasing tendency from 3 to 6 months; however, a significant decrease in both female (*p* = 0.005) and male patients (*p* < 0.001) was observed from 6 to 9 months (Fig. 3b). At 9 months, female and male patients with nAb titers > 40 were 58.3% (19/39) and 48.7% (28/48), respectively (Fig. 2b).

### Impact of age difference on nAb kinetics

The proportion of patients with high nAb titers decreased with time in both the < 50 and ≥ 50 years age groups (Fig. 2c). The GMT values for the < 50 years age group at three time points were 41.8, 33.3, and 24.7, whereas those for ≥ 50 years were 100.8, 92.2, and 49.5, respectively. Moreover, in both the age groups, no significant reduction was observed from 3 to 6 months (*p* = 1.000 and *p* = 0.266, respectively); however, nAb titers were significantly reduced from 6 to 9 months (*p* < 0.001 and *p* = 0.020, respectively) (Fig. 3c). Sex distribution (*p* = 0.382) and three visit time intervals (*p* > 0.050) showed no statistical differences in either group, except for the proportion of disease severity (*p* < 0.001). Of the total, 10 patients (25.6%) to report severe disease or above belonged to the ≥ 50 years age group, whereas none of them belonged to the < 50 years age group. Additionally, median nAb titers at the three time points were 40, 40, and 20 in the < 50 years age group, whereas those in the ≥ 50 years age group were 80, 80, and 40, respectively (Supplementary Table 1). At 9 months, the proportion of patients with nAbs over 40 were 33.3% (16/48) in the age group < 50 compared with 79.5% (31/39) in the age group ≥ 50 years. Patients with a nAb titer of zero were found in 2% (1/48) in the < 50 years group, but were not identified in the ≥ 50 years group (Fig. 2c).

### Impact of disease severity difference on nAb kinetics

Results revealed that the proportion of patients with high nAb titers was reduced after 9 months of COVID-19-related symptom onset or diagnosis in severe and above severity groups (Fig. 2d). The GMT values for the below severe group at the three time points were 54.3, 43.9, and 29.4, whereas those for severe and above groups were 171.5, 211.1, and 98.5, respectively. The nAb responses in below severe and severe and above groups showed no significant decrease from 3 to 6 months (*p* = 0.095 and *p* = 1.000, respectively); however, a significant decrease was observed from 6 to 9 months (*p* = 0.001 and *p* = 0.035, respectively) (Fig. 3e). Additionally, Patients 48.1% (37/77) and 100% (10/10) of the patients with nAb titers ≥ 40 at 9 months belonged to severe and severe and above disease severity groups, respectively. Moreover, only 1.3% (1/77) of the patients in the severe disease severity group showed an nAb titer of zero, whereas no such patient was identified in the severe and above group (Fig. 2d). The nAb response remained unchanged in below moderate and moderate and above severity groups from 3 to 6 months; however, a significant decrease was observed from 6 to 9 months (*p* = 0.006 and *p* = 0.001, respectively) (Fig. 3d). In addition, nAb titers in the below moderate and moderate and above severity groups were compared according to two age groups (< 50 and ≥ 50 years). In the ≥ 50 years age group, no significant nAb titer changes were observed in either below moderate or moderate and above groups (*p* > 0.050) from 3 to 6 months, whereas nAb titers were significantly reduced in both the groups (*p* < 0.001 for both) from 6 to 9 months (Fig. 3f). In the < 50 years age group, nAb titers remained unchanged in below moderate or moderate and above disease severity groups (*p* > 0.050) from 3 to 6 months. Furthermore, no significant reduction was found in the below moderate group (*p* = 0.156), whereas nAb titers significantly reduced in moderate and above disease severity groups from 6 to 9 months (*p* = 0.038) (Fig. 3g).

In the mild disease severity group (including 51 patients), no significant decrease in nAb titers was observed from 3 to 6 months; however, it decreased significantly from 6 to 9 months after COVID-19-related symptom onset or diagnosis (*p* < 0.001) (Supplementary Fig. 3a). Additionally, nAb titers in six asymptomatic patients showed a decreasing tendency from the first to third time points, but no significant reduction was reported over time until 9 months after COVID-19-related symptom onset or diagnosis (*p* = 0.610) (Supplementary Fig. 3b).

### Factors affecting nAb titers over time

Factors affecting the difference of nAb titers in a time-dependent manner were evaluated according to sex, age, and disease severity. The comparison of time (as a variable) with sex, age, and disease severity, resulted in *p*-values of 0.167, 0.188, and 0.081, respectively.

However, the differences in nAb titers based on the three above-mentioned factors were evaluated irrespective of time as well. Results demonstrated that the nAb titer was 1.46 times significantly higher in the ≥ 50 years than in the < 50 years age group (*p* = 0.036). Additionally, the nAb titer was 2.41 times higher in the moderate or above than in the below moderate disease severity group (*p* < 0.001). However, no significant differences in nAb titers were observed with respect to sex (*p* = 0.300).

## Discussion

Previous studies have demonstrated that nAb titers are relatively stable for at least 6 months in patients who recovered from mild-to-severe COVID-19^12,13^. In mild COVID-19 patients, a broad spectrum of the initial SARS-CoV-2-nAb response led to sustained antibodies for 10 months after infection^14^. Our study demonstrated variable but stable nAb titer at approximately 6 months after COVID-19 diagnosis; however, unlike the previous study, a more rapid decrease was observed from 6 to 9 months post-COVID-19 infection. Additionally, 3–6 months and the mild disease severity groups showed a significant decrease in nAb titers at approximately 9 months, whereas the asymptomatic group had a sustained nAb titer throughout.

According to a previous study, after a median of 58 days from the symptom onset, the median PRNT_50_ titer for SARS-CoV-2 was 40, and higher levels of neutralizing antibodies (with PRNT_50_ ≥ 320) were detectable in 12.6% of donors only^11^. Similar results were observed in our study, although the median was 109 days with PRNT_50_ as 40, and 7% of the total enrolled participants had PRNT_50_ ≥ 320.

According to our results, sex, age, and disease severity are the factors that could affect nAb titers^14–16^; however, these were not significant factors influencing nAb responses over time. The major strength of this study is that vaccination priority for recovered COVID-19 patients should be implemented regardless of sex, age, or severity.

A previous study demonstrated that a higher nAb titer was independently and significantly associated with males than with females^17^. Our findings suggest that nAb titers were higher in male than in female participants. However, a slower rate of decrease in the nAb titers was observed in females than in males, which was consistent with previous studies showing that the kinetics and plateau of female and male participants were similar when a longitudinal analysis was performed^18^ and that females had more stable antibody levels than males^14^. Regarding the effect of age on nAb kinetics, our results demonstrated that patients aged ≥ 50 years had higher median nAb titers at each visit time than those aged < 50 years; however, the latter showed relatively sustained nAb titers over time compared with the former, which was consistent with a previous study showing younger individuals with greater sustained antibody levels over time^14^.

According to previous studies, severely ill patients have been reported to have higher nAb titers^8,10^. Our findings showed similar results where moderate and above disease severity groups had higher nAb titers at all three visits compared with those from below moderate group. These results were observed in both the age groups (< 50 and ≥ 50 years).

Currently, SARS-CoV-2 variants are of great concern, and breakthrough vaccine infections with SARS-CoV-2 variants have been continuously identified globally^19–21^. Vaccinating uninfected and previously infected individuals is important for eliciting cross-variant nAbs production^22^. A previous study showed that the antibody response established by infection or vaccination effectively neutralizes SARS-CoV-2 B.1.1.7 (Alpha), but nAb titers against B.1.351 (beta), B.1.617.2 (delta), and P.1 (gamma) are significantly reduced^23^. Our findings also support the importance of vaccination, even in recovered COVID-19 patients, to maintain a high level of nAbs, thus reducing the risk of transmission and reinfection, particularly when emerging variants are of concern, as gradually decreased nAb activity was observed in recovered COVID-19 patients for less than one year. Our results suggest that vaccination should be conducted irrespective of sex, age, and disease severity identified during acute COVID-19 infection because these factors do not affect nAb titer persistency.

Despite significant findings, the present study had some limitations. We were unable to determine the initial status of the nAb titers in acute COVID-19 infected patients; therefore, the highest levels of nAb titers in the study participants could not be evaluated. However, previous studies have shown that nAbs reach a peak level at around 4 weeks and slowly decline afterward^24^. Next, we could not evaluate the impact of other medical treatments such as corticosteroids on nAb responses. A study reported that in immunomodulatory COVID-19-directed therapy, corticosteroids modulated antibody responses to decrease nAb titers, whereas azithromycin or hydroxychloroquine did not show a significant impact on it^25^. In our study, 88.5% of the participants did not require steroid treatment. Therefore, the impact of medical treatment is not expected to be high, even though azithromycin or hydroxychloroquine were frequently used in the COVID-19 outbreak in Korea in early 2020.

In summary, our results demonstrated that nAb titers showed variations for up to 6 months; however, a significant reduction was found over time. This response was also observed when the sex, age, and disease severity groups were compared separately. Furthermore, no differences in nAb reduction over time were identified when sex, age, and disease severity groups were compared separately. Unlike the other disease severity groups, the asymptomatic group showed a relatively low but sustained nAb titer over time. The potential for a higher nAb titer to be induced by vaccination suggests that reinfection could be further reduced by vaccinating patients who recovered from COVID-19. Determining the sequential nAb titers in recovered patients is important for predicting not only natural but vaccine-induced immunity against reinfection as well. Further studies on the kinetics and duration of nAb responses to SARS-CoV-2 are required to understand the key determinants of acquiring protection from reinfection and devise a proper vaccination strategy.

## Methods

### Study participants and design

Recovered COVID-19 patients were screened using the patient data registry from the Daegu Center for Infectious Diseases Control and Prevention. Patients diagnosed with COVID-19 using polymerase chain reaction (PCR) between February 19 and March 28, 2020, at Kyungpook National University Hospital, Daegu who provided informed consent to participate in the nAb tracking study were included. Those aged < 18 years and who could not make a voluntary decision to participate in the study were excluded. Patients were scheduled to visit the hospital every 3 months after the onset of the first COVID-19-related symptom or the date of COVID-19 diagnosis. First blood samples were collected from June 3–23, 2020 from 143 PCR-confirmed COVID-19-recovered patients. On the first day of the hospital visit, clinical characteristics were analyzed using an individualized questionnaire-based survey including details of demographics, clinical symptoms, admission information, COVID-19 diagnosis and symptom onset data, and disease severity during acute COVID-19. The second and third blood samples were collected from August 31–September 23 and November 27–December 22, 2020, respectively. Of the 143 patients, only those who underwent all three blood tests were selected for further analysis.

This study was approved by the Institutional Review Board (IRB) of Kyungpook National University Hospital (approval no.: 2020-05-004). All the participants provided informed consent through an IRB-approved form.

### Disease severity classification during acute COVID-19

COVID-19 clinical severity classification was performed as follows: (1) asymptomatic: patients tested positive for SARS-CoV-2 using a virologic test but had no COVID-19 symptoms; (2) mild illness: patients with any of the various signs and symptoms of COVID-19 but without shortness of breath, dyspnea, or abnormal chest imaging; (3) moderate illness: patients with evidence of lower respiratory disease during clinical assessment or imaging but did not require oxygenation other than room air; (4) severe illness: patients with evidence of lower respiratory disease during clinical assessment or imaging, and required oxygenation therapy (nasal prong, facial mask, or high-flow oxygen therapy); and (5) critical illness: patients with respiratory failure, septic shock, and/or multiple organ dysfunction, who required mechanical ventilation therapy, extracorporeal membrane oxygenation, or both.

### Neutralization assay

The SARS-CoV-2 of S clade (BetaCoV/Korea/KCDC03/2020, National Culture Collection for Pathogens (NCCP) 43326) was used for the neutralization activity analysis using the plaque reduction neutralization test (PRNT). Briefly, the antibody sample was diluted with phosphate-buffered saline (1:10) and serial dilution by two-folds. Diluted antibody samples (100 µL each) were mixed with the same volume of virus (100 plaque-forming units (PFU) in 100 µL) and incubated for 1 h at 37 °C. Subsequently, the whole reaction mixture was used to inoculate Vero cells for plaque assay. Virus-inoculated Vero cell plates (SPL Life Sciences, Pochen, Republic of Korea; NEST Scientific, #703003) were overlaid with agar and incubated at 37 °C in 5% CO_2_ for 3 days. The plates were then stained with crystal violet (Georgia Chemicals Inc., Norcross, GA, USA; Georgiachem, #548-6-29) to visualize the plaques formed by a non-neutralized infectious virus. The maximum dilution of serum to reduce the number of plaques by 50% of the serum-free virus was used to determine the amount of antibody present in the serum or the antibody effectiveness. This was denoted as the PRNT_50_ value. PBS served as negative and the serum yielding constant PRNT_50_ values during repeated measurements (Supplementary Fig. 1) served as positive controls. Each neutralization assay included positive and negative controls.

### Statistical analysis

Continuous variables were presented as medians and interquartile ranges (IQR), while categorical variables as numbers (percentage, %), and were compared using Fisher’s exact or chi-square test with Yates’ continuity correction. For non-normally distributed data, a nonparametric test was used. Changes in nAb titers at three time points were determined using a linear regression model. The nAb titers were graphed as geometric mean titers (GMT) with geometric standard error. Analysis of Variance (ANOVA) was used for the comparison of nAb titers between the groups. Paired t-tests were used to analyze the results of the first, second, and third blood samples, and p-values were corrected using Bonferroni's method. Additionally, a linear mixed model over time was used to compare the effects of sex, age, and disease severity. Statistical analysis was performed using SAS 9.4 and R version 4.0.3.

### Ethics statement

This study was reviewed and approved by the institutional review board of Kyungpook National University Hospital (approval no.: 2020-05-044). All methods were performed in accordance with the relevant guidelines and regulations.

## Supplementary Information

Supplementary Information.

## Data Availability

The data that support the findings of this study are available from the corresponding author, upon reasonable request.
