# Supplementary material for: Kinetics of neutralizing antibodies against SARS-CoV-2 infection according to sex, age, and disease severity
Source: Sci Rep. 2022 Aug 5;12:13491. doi: 10.1038/s41598-022-17605-1 (PMC9356129; doi:10.1038/s41598-022-17605-1)
Supplement: Supplementary file 1 — Supplementary Information. [file 41598_2022_17605_MOESM1_ESM.docx]

Kinetics of neutralizing antibodies against SARS-CoV-2 infection according to sex, age, and disease severity

Yoonjung Kim^1,*^, Joon-Yong Bae^2,*^, Kitae Kwon^1^, Hyun-Ha Chang^1^, Won Kee Lee^3^, Heedo Park^2^, Jeonghun Kim^2^, Isaac Choi^2^, Man-Seong Park^2,†^, Shin-Woo Kim^1,†^

^1^Department of Internal Medicine, Kyungpook National University Hospital, School of Medicine, Kyungpook National University, Daegu, Republic of Korea

^2^Department of Microbiology, Institute for Viral Diseases, College of Medicine, Korea University, Seoul, Republic of Korea

^3^Department of Medical Informatics, School of Medicine, Kyungpook National University, Daegu, Republic of Korea

* These are co-first authors who contributed equally to this work

†These authors are corresponding authors who contributed equally to this work

**Address for Correspondence**:

Shin-Woo Kim, MD, PhD

Department of Internal Medicine, Kyungpook National University School of Medicine,

130, Dongdeok-ro, Jung-gu, Daegu, 41944, Republic of Korea

Tel: 82-53-200-6525

Fax: 82-53-424-5542

E-mail: [ksw2kms@knu.ac.kr](mailto:ksw2kms@knu.ac.kr)

**Supplementary Table 1.** Clinical characteristics of the study population according to the age <50 and ≥50 group

|  | Age < 50 years  (n = 48) | Age ≥ 50 years  (n = 39) | Total  (n = 87) | *P*-value |
| --- | --- | --- | --- | --- |
| Age, median [IQR], years | 37.0 [25.5;41.0] | 60.0 [57.0;63.5] | 48.0 [36.0;60.0] | < 0.001 |
| Sex, n (%) |  |  |  |  |
| Male | 19 (39.6%) | 20 (51.3%) | 39 (44.8%) | 0.382 |
| Female | 29 (60.4%) | 19 (48.7%) | 48 (55.2%) |  |
| Disease severity, n (%) |  |  |  | < 0.001 |
| Asymptomatic | 6 (12.5%) | 0 (0.0%) | 6 (6.9%) |  |
| Mild | 36 (75.0%) | 15 (38.5%) | 51 (58.6%) |  |
| Moderate | 6 (12.5%) | 14 (35.9%) | 20 (23.0%) |  |
| Severe | 0 (0.0%) | 7 (17.9%) | 7 (8.0%) |  |
| Very severe | 0 (0.0%) | 3 (7.7%) | 3 (3.5%) |  |
| Height, median [IQR], cm | 167.0 [158.5;172.0] | 163.0 [156.5;170.0] | 164.0 [158.0;172.0] | 0.187 |
| Weight, median [IQR], kg | 64.0 [54.0;74.5] | 64.0 [57.0;70.0] | 64.0 [55.0;71.5] | 0.993 |
| First time (days) | 112.0 [105.5;116.0] | 110.0 [105.5;116.0] | 111.0 [105.5;116.0] | 0.806 |
| Second time (days) | 81.0 [78.0;85.0] | 78.0 [76.0;84.5] | 81.0 [76.0;85.0] | 0.310 |
| Third time (days) | 92.0 [92.0;95.0] | 92.0 [91.0;92.0] | 92.0 [91.5;93.5] | 0.113 |
| Neutralizing antibody titers, median [IQR] |  |  |  |  |
| First time | 40.0 [20.0;80.0] | 80.0 [40.0;160.0] | 80.0 [40.0;80.0] | < 0.001 |
| Second time | 40.0 [20.0;80.0] | 80.0 [40.0;160.0] | 40.0 [20.0;80.0] | < 0.001 |
| Third time | 20.0 [20.0;40.0] | 40.0 [40.0;80.0] | 40.0 [20.0;80.0] | < 0.001 |
| ICU admission, n (%) |  |  |  |  |
| No | 48 (100.0%) | 35 (89.7%) | 83 (95.4%) | 0.079 |
| Yes | 0 (0.0%) | 4 (10.3%) | 4 (4.6%) |  |
| Oxygen treatment, n (%) |  |  |  | 0.001 |
| No | 48 (100.0%) | 29 (74.4%) | 77 (88.5%) |  |
| Yes | 0 (0.0%) | 10 (25.6%) | 10 (11.5%) |  |
| MV use, n (%) |  |  |  | 0.172 |
| No | 48 (100.0%) | 36 (92.3%) | 84 (96.6%) |  |
| Yes | 0 (0.0%) | 3 (7.7%) | 3 (3.4%) |  |
| ECMO use, n (%) |  |  |  | 0.917 |
| No | 48 (100.0%) | 38 (97.4%) | 86 (98.9%) |  |
| Yes | 0 (0.0%) | 1 (2.6%) | 1 (1.1%) |  |
| DM, n (%) |  |  |  | 0.001 |
| No | 47 (97.9%) | 28 (71.8%) | 75 (86.2%) |  |
| Yes | 1 (2.1%) | 11 (28.2%) | 12 (13.8%) |  |
| HTN, n (%) |  |  |  | < 0.001 |
| No | 46 (95.8%) | 24 (61.5%) | 70 (80.5%) |  |
| Yes | 2 (4.2%) | 15 (38.5%) | 17 (19.5%) |  |
| CKD, n (%) |  |  |  | 0.172 |
| No | 48 (100.0%) | 36 (92.3%) | 84 (96.6%) |  |
| Yes | 0 (0.0%) | 3 (7.7%) | 3 (3.4%) |  |
| Liver disease, n (%) |  |  |  | 0.124 |
| No | 47 (97.9%) | 34 (87.2%) | 81 (93.1%) |  |
| Yes | 1 (2.1%) | 5 (12.8%) | 6 (6.9%) |  |
| Hematologic malignancy, n (%) |  |  |  | NA |
| No | 48 (100.0%) | 39 (100.0%) | 87 (100.0%) |  |
| Yes | 0 (0.0%) | 0 (0.0%) | 0 (0.0%) |  |
| Solid tumor, n (%) |  |  |  | NA |
| No | 48 (100.0%) | 39 (100.0%) | 87 (100.0%) |  |
| Yes | 0 (0.0%) | 0 (0.0%) | 0 (0.0%) |  |
| CVA, n (%) |  |  |  | 0.385 |
| No | 48 (100.0%) | 37 (94.9%) | 85 (97.7%) |  |
| Yes | 0 (0.0%) | 2 (5.1%) | 2 (2.3%) |  |
| COPD, n (%) |  |  |  | 1.000 |
| No | 48 (100.0%) | 39 (100.0%) | 87 (100.0%) |  |
| Yes | 0 (0.0%) | 0 (0.0%) | 0 (0.0%) |  |
| Heart disease, n (%) |  |  |  | 1.000 |
| No | 47 (97.9%) | 38 (97.4%) | 85 (97.7%) |  |
| Yes | 1 (2.1%) | 1 (2.6%) | 2 (2.3%) |  |

Abbreviations: IQR, interquartile range (range from lower quartile to upper quartile); ICU, intensive care unit; ECMO, extracorporeal membrane oxygenation; MV, mechanical ventilator; DM, diabetes mellitus; HTN, hypertension; CKD, chronic kidney disease; CVA, cerebrovascular accident; COPD, chronic obstructive pulmonary disease. Heart diseases (heart failure, arrhythmia, valvular heart disease, and coronary heart disease); NA, not available; *p*-value was obtained using a chi-square test with Yates continuity correction.

**Supplementary Figure 1. Evaluation of PRNT50 titers using positive control serum (n = 7)**


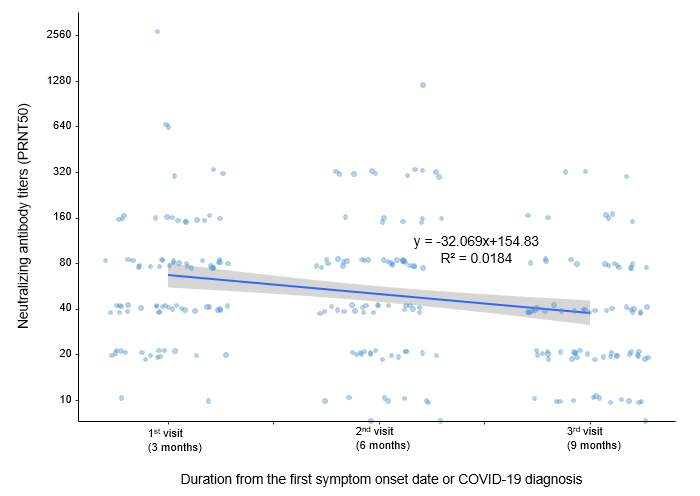


Supplementary Figure 2. Changes of neutralizing antibody (nAb) titers at three different time points. A linear regression model showed a decreasing tendency of neutralizing antibodies (nAbs) over time.

(A)

(B)

**Supplementary Figure 3. Variations of neutralizing antibody (nAb) titers over time in mild disease severity and asymptomatic groups.** Titers are graphed as geometric mean titers (GMT) with geometric standard error. Blood samples were initially collected 3 months post-COVID-19 symptom onset or diagnosis and were then followed-up for additional blood collections at 6 and 9 months after symptom onset of COVID-19 or diagnosis (a) NAb titers of 51 mild disease severity group individuals. (b) NAb titers of six asymptomatic disease severity group individuals. The *p*-value was obtained using a Bonferroni correction of the post hoc comparisons after repeated measures analysis of variance.
